# Mechanisms and Pharmacotherapy for Ethanol-Responsive Movement Disorders

**DOI:** 10.3389/fneur.2020.00892

**Published:** 2020-08-25

**Authors:** Jingying Wu, Huidong Tang, Shengdi Chen, Li Cao

**Affiliations:** Department of Neurology and Institute of Neurology, Rui Jin Hospital, Shanghai Jiao Tong University School of Medicine, Shanghai, China

**Keywords:** ethanol, movement disorder, GABA receptor, low-voltage-activated calcium channel, glutamate receptor

## Abstract

Ethanol-responsive movement disorders are a group of movement disorders of which clinical manifestation could receive significant improvement after ethanol intake, including essential tremor, myoclonus-dystonia, and some other hyperkinesia. Emerging evidence supports that the sensitivity of these conditions to ethanol might be attributed to similar anatomical targets and pathophysiologic mechanisms. Cerebellum and cerebellum-related networks play a critical role in these diseases. Suppression of inhibitory neurotransmission and hyper-excitability of these regions are the key points for pathogenesis. GABA pathways, the main inhibitory system involved in these regions, were firstly linked to the pathogenesis of these diseases, and GABA_A_ receptors and GABA_B_ receptors play critical roles in ethanol responsiveness. Moreover, impairment of low-voltage-activated calcium channels, which were considered as a contributor to oscillation activity of the nervous system, also participates in the sensitivity of ethanol in relevant disease. Glutamate transporters and receptors that are closely associated with GABA pathways are the action sites for ethanol as well. Accordingly, alternative medicines aiming at these shared mechanisms appeared subsequently to mimic ethanol-like effects with less liability, and some of them have achieved positive effects on different diseases with well-tolerance. However, more clinical trials with a large sample and long-term follow-ups are needed for pragmatic use of these medicines, and further investigations on mechanisms will continue to deepen the understanding of these diseases and also accelerate the discovery of ideal treatment.

## Introduction

Ethanol is known to have a significant influence on human bodies, especially on the nervous system (1). Despite those negative effects as described in various studies, patients with certain diseases benefited from the consumption of ethanol. The clinical manifestation of ethanol-responsive movement disorders (ERMDs) could be significantly improved after ethanol intake. Patients with essential tremor (ET), one of the most common movement disorders influencing ~1% of the population worldwide (2), were first reported to respond to ethanol in 1949 by Critchley (3). Similarly, alcohol intake has long been known to decrease myoclonic symptoms in most myoclonus dystonia (MD) patients since 1967 (4). Besides, cases of different phenotypes of dystonia (5–7), dyssynergia cerebellaris myoclonica (8–11), epilepsia partialis continua (12), post-hypoxic myoclonus (13), and tremor with multiple sclerosis (14) are also reported to have similar positive responses to ethanol (Table 1).

**Table 1:** Effects of ethanol on reported ethanol-responsive movement disorders.

**Diseases**	**Year of first report**	**Proportion of ethanol responders**	**Number of reported cases**	**Administration route**	**Effective dose**	**Clinical effects**	**References**
**Tremor**							
Essential tremor	1949	74%	–	Intra-arterial/Oral	Small dose	Decrease tremor amplitude; reduce gait disturbances	(3, 15–17)
Tremor with multiple sclerosis	2008	–	1	Oral	–	Decrease tremor amplitude	(14)
**Myoclonus**							
Myoclonus dystonia	1967	77%	–	Oral	–	Reduce myoclonic symptoms; improve cerebellar learning deficit	(4, 18, 19)
Dyssynergia cerebellaris myoclonica	1990	–	12	Oral	30–40 g/180–200 ml spirit	Reduce myoclonic symptoms; attenuate the giant cortical SEPs	(8–11)
Post-hypoxic myoclonus	1991	–		Oral	12.6 g	Reduce myoclonic symptoms	(13)
Epilepsia partialis continua	2014	–	1	Oral	–	Reduce myoclonic symptoms	(12)
Action myoclonus in prostate cancer	2015	–	2	Oral	Small dose	Reduce myoclonic symptoms	(20)
**Dystonia**		29%					(21)
Isolated dystonia	1993	–	1	Oral	60 g	Reduce myoclonic symptoms; improve myorrhythmic movements	(5)
Spasmodic dysphonia	2015	56%	–	Oral	2 drinks	Reduce myoclonic symptoms	(6)
Writer's cramp	2012	–	1	Oral	200 ml beer	Reduce myoclonic symptoms	(7)

In spite of the differences in phenomenology of all these diseases mentioned above, their responsiveness to ethanol sets them apart from other movement disorders and brings up the possibility that the pathogenesis of these conditions might be linked to shared anatomical networks and the sensitivity to ethanol might be attributed to related mechanisms. Though not yet explored thoroughly, some hypotheses have already been raised to explain the phenomenon, mostly involving the GABA system, low-voltage-activated calcium channels, and glutamate pathways. Nevertheless, further investigations are needed to elucidate the pathophysiology.

Ethanol therapy has been already applied to some ethanol-responsive diseases such as essential tremor with clinical results. However, the therapeutic use of ethanol is limited by side effects involving the liver and brain and a high rate of alcoholism (22). Therefore, with deeper understanding of the pathogenesis, corresponding medicines have emerged subsequently to mimic alcohol-like effects through the common pathways among ERMDs.

In this review, we will elaborate reported diseases with ethanol responsiveness and analyze their shared anatomical networks, summarize the possible mechanisms underlying the treatment-like effects of ethanol, describe the main deficiencies of current ethanol therapy, and then introduce progress on relevant medication that could substitute for ethanol to avoid some of its side effects.

## Anatomical Networks Involved in Relevant Diseases

Ingestion of ethanol has long been proved to have a treatment-like influence on hyperkinetic movement disorders (Table 1). While the therapeutic effects vary among different diseases, shared anatomical networks in these diseases, especially cerebellum and neural circuits related to the cerebellum, suggest the possibility of common mechanisms underlying ethanol responsiveness.

### Cerebellum and ERMDs

As a sophisticated brain region that performs a wide range of crucial roles in movement disorders, the cerebellum integrates information from the spinal cord, cerebral cortex, and vestibular nuclei; compares efference copies and reafference signals; and corrects for discrepancies between them to enable the execution of smooth, well-coordinated movements. Undoubtedly, evidence from most published studies indicates the critical role of the cerebellum in the pathophysiology of ERMDs.

Essential tremor (ET), the most common movement disorder worldwide (2), is predominantly related to the cerebellum and is mainly linked to Purkinje cells, the main cerebellar output, and the inhibitory neurons in the cerebellar cortex (23). Decreased density (24, 25), increased heterotopic rates (26), morphological changes on dendritic arborizations (27), and axonal changes (28) of Purkinje cells could be referred to hyperactivity of the cerebellum and consequently to tremor. Besides, neuroimaging studies, as a non-invasive approach of research, have also highlighted the morphologic abnormalities in the cerebellum. Atrophic changes in different lobules of the cerebellum, including gray matter and white matter, are revealed in previous studies (29, 30).

Myoclonus-dystonia being the second common disease in ERMDs, ~76.9% of reported patients of myoclonus-dystonia (MD) were responsive to ethanol (31). Besides myoclonic symptoms, cerebellar learning deficits could also get improved *via* ethanol intake since reduced baseline acquisition of conditioned eyeblink responses and normal blink reflex recovery cycle were observed in MD patients after drinking (19), providing clinical evidence for the possible role of the cerebellum. Neurophysiological (32), structural (33), functional (34), and metabolic (35) studies also support the cerebellum as the subcortical generator underlying motor symptoms in MD. Moreover, impaired motor learning and abnormal nuclear envelope in the cerebellar Purkinje cells were detected in MD mouse models, the paternally inherited *Sgce* heterozygous knockout mice (36), and acute cerebellar knockdown of *Sgce* could reproduce salient features of MD in mice (37), further confirming the essential role of cerebellum in this disease.

As for dystonia, animal studies have also provided compelling evidence of a role played by the cerebellum in generating dystonic-like movements and postures, including altered burst patterns in Purkinje cell firing (38, 39), dysfunctional interactions with basal ganglia, another essential region for dystonia (40), and abnormal morphology of Purkinje cells (41). Studies on clinical patients with dystonia have provided neuropathologic (42), neurophysiologic, and functional neuroimaging evidence (43) for the significance of the cerebellum in dystonia.

In addition, though not compelling enough, other ERMDs are also likely linked to the cerebellum, revealed by different aspects of studies. For example, Ganos et al. (44) proposed that the cerebellum also played a critical role in the pathophysiology of cortical myoclonus including dyssynergia cerebellaris myoclonica (DCM) based on alterations in inhibitory neurotransmission and the presence of cerebellar pathology.

Overall, there is little doubt about the relationship between the cerebellum and ERMDs, and it is more and more likely that neural circuits and pathophysiological mechanisms involving the cerebellum are of great significance for these diseases.

### Cerebellum-Related Networks in ERMDs

#### Cerebello-thalamo-Cortico-Cerebellar Loop

Notably, given the dearth of direct connections between the cerebellum and peripheral nervous system, the intricate task of the cerebellum is mainly accomplished by modulating the excitability of the primary motor cortex through the cerebello-thalamo-cortical tract. The altered cerebello-thalamo-cortico-cerebellar loop could be detected in different ERMDs.

Electrophysiology, structural magnetic resonance imaging (MRI), diffusion MRI, and positron emission tomography (PET) studies have revealed various abnormalities in cerebello-thalamo-cortico-cerebellar circuits in ET (45, 46). Based on that, a recent study revealed the distinctive white matter microstructural values localize to the cerebellar peduncles and thalamo-cortical visual pathways *via* diffusion tensor imaging (DTI) and fractional anisotropy, suggesting that a cerebello-thalamo (posterior) cortical network rather than a cerebello-thalamo-motor cortical network takes part in ET (47).

Functional MRI (fMRI) was performed using a validated “Go/No go” task to assess the possible network causing MD and demonstrated a distinct association of motor symptoms in MD with the cerebello-thalamo-cortical system (48). A study with voxel-based morphometry and DTI also illustrated the white matter changes found in the subthalamic area of the brain stem, connecting the cerebellum with the thalamus, which are compatible with the hypothesis that abnormal function in MD involves cerebello-thalamo-cortical pathways. Moreover, an altered cerebello-thalamo-cortico-cerebellar loop was revealed in other phenotypes of dystonia including MD through functional imaging (49) and neurophysiologic studies (50, 51).

In addition, abnormalities of key structures within the cerebello-thalamic-cortico-cerebellar loop were unveiled in some other ERMDs, despite lack of evidence of functional connectivity. Degeneration of cerebellar and thalamic regions was the pathological substrates for tremor in multiple sclerosis patients, implicated by a study based on structural MRI (52). Decrease in amplitude of giant cortical somatosensory-evoked potentials (SEPs) was confirmed in patients with DCM after alcohol ingestion (10), indicating the possible role of the cerebral cortex. Further studies based on neuroimaging and electrophysiology are needed to elucidate the connection between these regions.

#### Guillain–Mollaret Triangle

The Guillain–Mollaret triangle means the loop from the dentate nucleus to the red nucleus to inferior olivary (IO) nucleus to the dentate nucleus *via* the cerebellar cortex, which also participates in the pathogenesis of ERMDs such as ET and MD.

IO neurons have a natural tendency to oscillate in a synchronous pattern at a frequency of 4–10 Hz, which is exactly the most common frequency seen in ET patients (53). Boecker et al. (54) once used ${\text{H}}_{2}^{15}$O PET to investigate the effect of ethyl alcohol on regional cerebellar blood flow in patients with alcohol-responsive ET and found that alcohol suppressed cerebellar activities in both control and ET patients but induced increasing activities in IO only in the latter group. Animal models are also useful to interrogate the pathogenesis of the diseases. Harmaline is most frequently used to induce tremor in experimental models. It can induce ET-like tremor through its actions on the IO nuclei. Like tremor in ET patients, harmaline-induced tremor could be suppressed by ethanol (55). Besides, studies of diffusion tensor image (DTI) indicated the involvement of the superior cerebellar peduncle fiber tracts in ET patients, which receive input from the dentate nucleus and send output to the red nucleus (56). Based on these findings, a disturbance in the Guillain–Mollaret triangle may underlie tremor pathogenesis in ET.

As for MD patients, besides the involvement of the cerebello-thalamo-cortico-cerebellar pathway, increased white matter volume and fractional anisotropy and decreased mean diffusivity were also found in the subthalamic area of the brain stem, including the red nucleus (33), also indicating the possible role of Guillain–Mollaret triangle.

## Pathophysiologic Mechanisms

Since the discovery of ethanol responsiveness, researchers have never stopped investigating the pathophysiologic mechanisms underlying the phenomena. However, the specific mechanisms have not yet been fully elucidated. As mentioned above, ERMDs share common anatomical networks such as cerebellum and cerebellum-related circuits, which means the therapeutic effects of ethanol are largely attributed to similar pathways that play a critical role in these regions. Ethanol responsiveness might be the result of the combination of some of these known mechanisms or some other unknown pathways.

### GABA Pathways and ERMDs

As the main inhibitory system in the nervous system, especially in cerebellum-related circuits, the reduction of GABAergic inhibitory function contributes to various ERMDs, such as ET, MD, progressive myoclonic epilepsy (44), and dystonia (57). Thus, GABA pathways were first linked to ethanol responsiveness, in which GABA receptors, including GABA_A_ receptors and GABA_B_ receptors, play a critical role.

#### GABA_A_ Receptors and ERMDs

**Figure 1:**
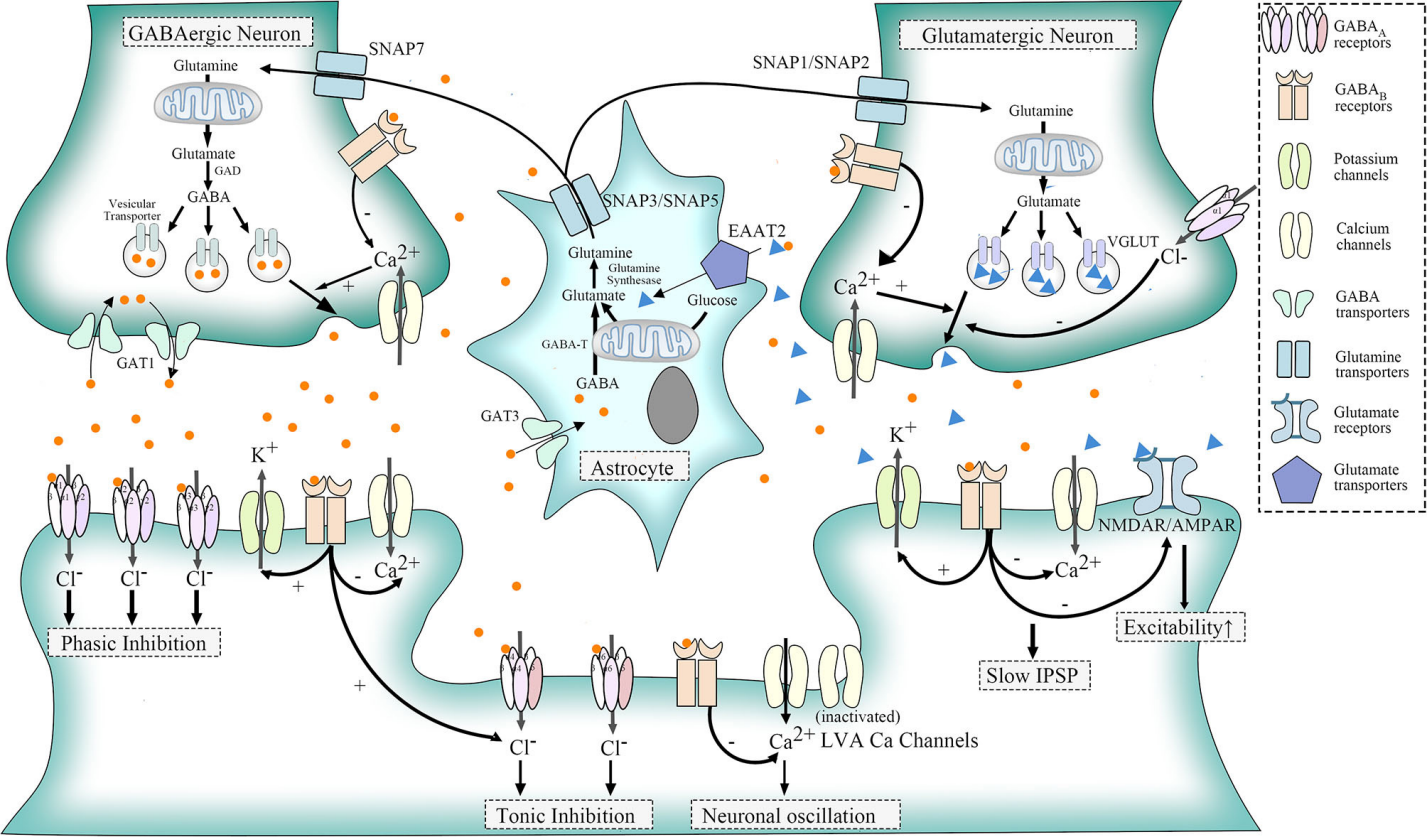
Physiologic functions of GABA receptors, LVA Ca^2+^ channels, and glutamatergic pathways. GABA, transformed from glutamate in GABAergic neurons, acts through the combination of specific receptors. GABA_A_ receptors belong to ligand-gated chloride (Cl-) channels. Postsynaptic GABA_A_ receptor, a mediator for phasic inhibition, consists of two α(α1–α3) subunits, two β subunits, and one γ2 subunit. The extra-synaptic GABA_A_ receptor, however, elicits tonic inhibition, containing two α (α4, α6) subunits, two β subunits, and one δ subunit. GABA_B_ receptors could be distributed in three different sites. When located presynaptically, they could regulate the release of neurotransmitters of GABAergic and glutamatergic neurons *via* the suppression of HVA calcium channels. As for postsynaptic GABA_B_ receptors, they could induce slow IPSP by activating outward potassium channels and suppressing inward HVA calcium channels. They also inhibit NMDAR/AMPAR to counteract the excitatory influence of glutamate. Besides, extra-synaptic GABA_B_ receptors, as well as other G protein-coupled receptors, could activate LVA Ca channels to induce neuronal oscillation, though part of LVA calcium channels normally remain silent. With regard to glutamine, which could be transported into GABAergic neurons and glutamatergic neurons *via* SNAP7 and SNAP1/SNAP2, respectively, are the basic materials for synthesis of glutamate. In addition, astrocytes could uptake GABA through GABA transporter 3 (GAT3) and glutamate *via* EAAT2. Both glutamate and GABA could change into glutamine in astrocytes, and glutamine will be released to the intercellular space again through SNAP3/SNAP5.

GABA_A_ receptors (GABA_A_Rs) are one of ligand-gated chloride channels. According to distribution, these receptors are divided into three types: postsynaptic GABA_A_R containing 2 α1/α2/α3 subunits, 2 β subunits, and 1 γ2 subunit that respond to benzodiazepine but not to ethanol; extra-synaptic expressed GABA_A_R which contains 2 α4/α6 subunits, 2 β subunits, and 1 δ subunit that respond to ethanol, general anesthetics, and neurosteroids rather than benzodiazepine (58, 59); and presynaptic GABA_A_Rs located on parallel fibers which could depolarize terminals to induce glutamate release onto molecular layer interneurons and Purkinje cells and thereby lead to increased excitability of target neurons (60) (Figure 1).

The connection between GABA_A_R and ERMDs is controversial. Postsynaptic GABA_A_Rs are first considered as a candidate participant in the pathogenesis of essential tremor (ET). Not only α1 subunit-deficient mice manifest with similar postural and kinetic tremor to ET (61), but PET studies (62) and autoradiography experiments (63) also supported altered postsynaptic GABA_A_R functions among ET patients. However, the inconsistency in manifestations between α1^−/−^ mice and ET patients casts doubt in this assumption. Specifically, tremor in α1^−/−^ mice presents earlier in life and has a higher mean frequency (19.3 Hz), accompanied by considerable incoordination, which is not typical for ET patients (61). Besides, there is no report about any association between homozygous α1 subunit mutations and patients with ET or other ERMDs, shedding the possibility that loss-of-function of α1 subunits only share part of mechanisms with ET pathogenesis.

**Figure 2:**
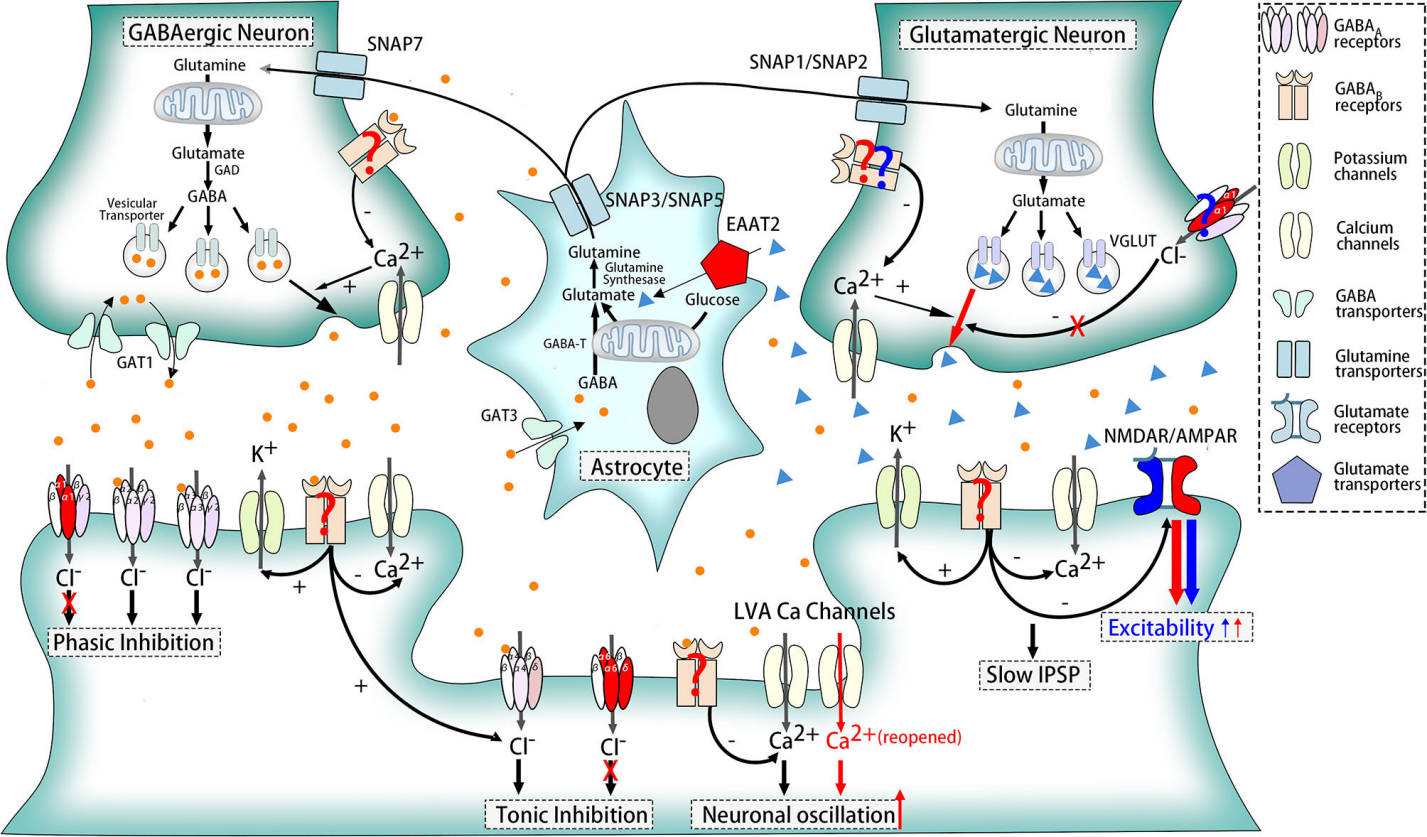
Pathophysiologic mechanisms of ethanol-responsive movement disorders. Dysfunction of receptors and transporters marked in red participates in the pathogenesis of essential tremor, including α1, δ, and α6 subunits of GABA_A_Rs, EAAT2, reopened LVA Ca^2+^ channels, and AMPAR. GABA_B_Rs are also the potent participants, which was indicated in red question marks. Besides, compositions marked in blue contribute to myoclonus dystonia such as NMDAR and AMPAR. GABA_A_Rs and GABA_B_Rs in glutamatergic neurons are also likely to play a role in MD, shown in blue question marks as well.

Based on that, we try to explain it in three different angles. (1) Postsynaptic GABA_A_R alterations are only restricted to one or certain parts of oscillation circuitry in ERMDs. For example, defective GABA_A_R were detected in the dentate nucleus rather than the cerebellar cortex in patients with ET (64). (2) Presynaptic rather than postsynaptic GABA_A_Rs play a major role in the pathogenesis. In fact, the *in vivo* PET study of ET patients reveals increased benzodiazepine antagonist [11C]flumazenil signals (62) while *ex vivo* autoradiography experiments found decreased benzodiazepine binding (63). (3) Extra-synaptic GABAARs, which do not contain α1 subunits, also contribute to the mechanisms. Besides, α1^−/−^ mice, δ^−/−^, and α6^−/−^ mice also exhibited similar tremor to ET, and their symptoms were significantly improved after injection of inhibitors of extra-synaptic GABA_A_ receptors (65) (Figure 2).

**Figure 3:**
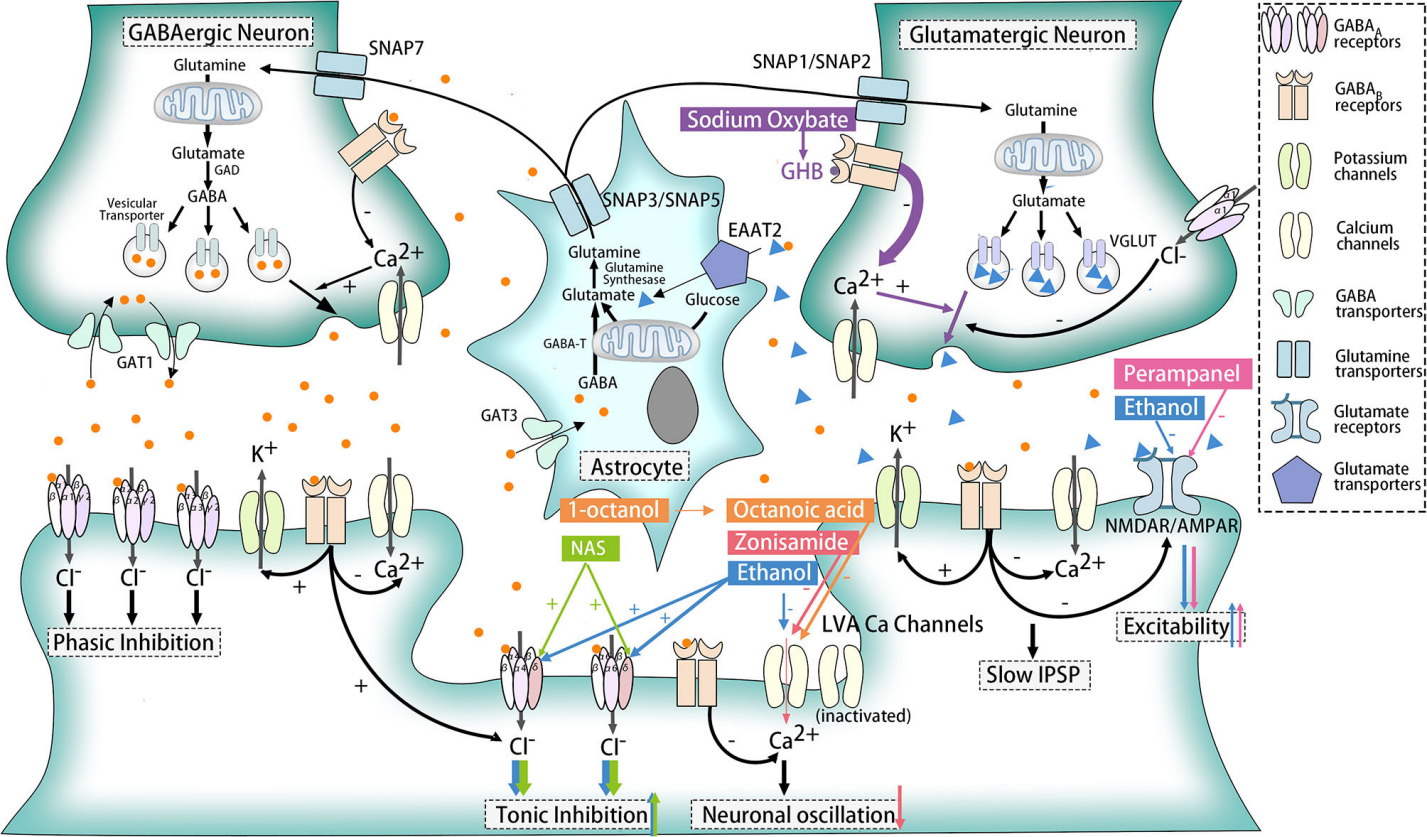
Effects of ethanol and other medicines on ethanol-responsive movement disorders. The action sites of ethanol, sodium oxybate, NAS, zonisamide, perampanel, 1-octanol, and octanoic acid are marked with blue, purple, green, red, pink, and orange, respectively. Ethanol serves as an activator for the δ subunit of GABA_A_Rs and an inhibitor for LVA Ca^2+^ channels, NMDAR and AMPAR. Sodium oxybate could convert into GHB, a structural analog of GABA for GABA_B_ receptors. NAS is also the agonist of the δ subunit of GABA_A_Rs, while zonisamide works as an antagonist for LVA Ca^2+^ channels. As for perampanel, it could suppress AMPAR selectively. Besides, 1-octanol and its active metabolite octanoic acid exhibited its ability to block LVA Ca^2+^ channels, but other mechanisms might participate at the same time.

Ethanol is one of the activators of δ subunits of extra-synaptic GABA_A_ receptors (66, 67). Tonic inhibition *via* extra-synaptic GABA_A_ receptors is critical for long-term maintenance of the inhibitory status of neurons. Thus, ethanol might enhance tonic inhibition of target cells to compensate for dysfunction of postsynaptic or presynaptic GABA_A_Rs and thereby relieve symptoms in ERMDs (Figure 3).

#### GABA_B_ Receptors and ERMDs

GABA_B_ receptors (GABA_B_Rs), belonging to G protein-coupled receptors, perform different functions according to their location. When located presynaptically, activated GABA_B_Rs prevent the release of neurotransmitters like GABA and glutamate. Postsynaptic GABA_B_Rs, however, could induce hyperpolarization and slow inhibitory postsynaptic potentials (IPSP) and suppress glutamate receptors as well. Still, some GABA_B_Rs exist extra-synaptically to inhibit T-type calcium channels, which will be further elaborated later (68) (Figure 1). The relationship between GABA_B_R and ERMDs is unclear, although abnormality of GABA_B_R was detected in the dentate nucleus of ET patients (64) (Figure 2). Ethanol, as an activator for presynaptic GABA_B_ receptors, is able to inhibit the release of glutamate and thereby suppress the excitability of postsynaptic cells (69), which help alleviate hyperkinetic symptoms of ERMDs (Figure 3).

### Low-Voltage-Activated Calcium Channels and ERMD

Low-voltage-activated (LVA) calcium (Ca) channels, known as T-type Ca^2+^ channels, belong to voltage-gated Ca^2+^ channels with high-voltage-activated (HVA) Ca^2+^ channels and intermediate-voltage-activated (IVA) Ca^2+^ channels. They are activated by G protein-coupled receptors such as GABA_B_Rs. Normally, the opening of HVA Ca^2+^ channels needs a large membrane depolarization, while a weak depolarization near the resting membrane potential could trigger the LVA Ca^2+^ channels, with IVA in between. Part of LVA Ca^2+^ channels stay inactivated under normal circumstances (70) (Figure 1).

LVA Ca^2+^ channels are confirmed to be associated with controlling neuronal excitability and oscillatory behavior, and an increasing number of studies have proved its relation with repetitive burst discharges. Genetically, the *HS1BP3* gene, which encodes HS1-binding protein 3, is one of candidate genes for familial essential tremor (71) and highly expressed in motor neurons and Purkinje cells regulating Ca^2+^-dependent protein kinase activation of tyrosine and tryptophan hydroxylase (72). As for animal experiments, lack of 4–10 Hz rhythmic burst discharges in inferior olive (IO), a crucial promoter for essential tremor, was present in mice lacking the Ca_V_3.1 gene (73), and five T-type calcium antagonists, including ethosuximide and zonisamide, suppressed tremor in two different animal tremor models (74), suggesting that LVA Ca^2+^ channels are the molecular pacemaker substrates for intrinsic neuronal oscillations of IO neurons, and this mechanism is likely to be a pathological cause of essential tremor (Figure 2).

Acute ethanol administration has been shown to cause disruption of native T-currents, and long-term disruption of LVA Ca^2+^ channel expression and function occurs upon withdrawal after chronic intermittent ethanol exposures (75, 76). Ethanol inhibition of LVA Ca^2+^ channels is due to activation of the protein kinase C pathway, with a major effect on the hyperpolarized shift in inactivation (Figure 3). Among three isoforms of T-type Ca^2+^ channels (Ca_V_3.1, Ca_V_3.2, Ca_V_3.3), Ca_V_3.2 was significantly affected by ethanol, and might be another novel target for ethanol (77).

### Glutamate Pathways and ERMDs

Impairment of glutamate pathways, especially different glutamatergic receptors, contributes to the onset of ERMDs such as ET and MD (78). One candidate alternation is the glutamate transporter mainly located in astrocytes, excitatory amino acid transporter 2 (EAAT2). These receptors uptake glutamate into astrocytes to regulate the concentration of glutamate in the extracellular space (Figure 1). Involvement of EAAT2 is supported by postmortem and *ex vivo* experimental studies that revealed decreased EAAT2 in the cerebellar cortex and increased expression in the thalamus (79, 80) in patients with ET. In the genetic aspect, one variant (rs3794087) of the *SLC1A2* gene encoding EAAT2 seems to be related with essential tremor (81, 82), though some other studies doubted this association (83–85). N-Methyl-D-aspartate receptor (NMDAR), a postsynaptic glutamate receptor and a regulator of efflux of N-acetylaspartate (NAA) (86), is another possible participant in pathogenesis. Decreased NAA/creatine and NAA/choline ratios in the cerebellum, although no difference was observed in thalamus (80) or basal ganglia (87), backed up this view. In addition, another postsynaptic and extra-synaptic glutamate receptor, α-amino-3-hydroxy-5-methyl-4-isoxazolepropionic acid receptor (AMPAR), also plays a significant role, supported by success suppression by AMPAR antagonist of harmaline-induced tremor, one of classic animal models for ET (88) (Figure 2).

Moreover, glutamate pathways are closely associated with GABA systems so that alterations in GABA receptors will also have significant influence on glutamate transmission. Glutamine, which could be transported into GABAergic neurons and glutamatergic neurons *via* system N receptors, SNAP7 and SNAP1/SNAP2, respectively, are the basic material to the synthesis of glutamate. GABA, subsequently, could be transformed into GABA in GABAergic neurons. To maintain a balance concentration in the intercellular space, astrocytes then take the responsibility to uptake GABA through GABA transporter 3 (GAT3) and glutamate *via* EAAT2. Both glutamate and GABA could change into glutamine again in astrocytes, and glutamine will be released to the intercellular space again through another two system *N* receptors SNAP3/SNAP5. In addition, presynaptic and postsynaptic GABA receptors in glutamatergic synapses could inhibit the release of glutamate when activated (Figure 1). Impairment of this relation is a potential mechanism for MD. MD is often caused by mutations in the *SGCE* gene (89). *SGCE* encodes ε-sarcoglycan (ε-SG) and a brain-specific isoform, expressed in GABA postsynaptic and presynaptic cells, respectively, and loss-of-function mutations are only found in those parts related to ε-SG (90). Meanwhile, long-term depression of glutamatergic synapses was shown to be inhibited in a myoclonus dystonia mouse model (78). Therefore, dysfunction of postsynaptic GABA receptors might result in the pathogenesis of MD (Figure 2).

Ethanol is able to antagonize the effect of harmaline through impairment of NMDA-mediated glutamate transmission (91). Downregulation of EAAT2 and AMPAR (92) among chronic alcohol assumption also suggests their possible role for ethanol responsiveness (Figure 3), but more studies are in need for confirm their functions.

## Progress on Pharmacotherapy for ERMDs

Though ethanol is able to alleviate symptoms of ERMDs to some degree, researchers gradually found that concomitant with improvements, the consumption of ethanol can also bring about a host of problems, regarding its efficacy, adverse effects, and misuse. First of all, ethanol is rapidly metabolized and eliminated in the human's body and exhibits a tendency to produce a rebound of involuntary movements when it wears off (22). These characteristics make it nearly impossible for ethanol to serve as long-term control or modulation of the frequency of paroxysms. Furthermore, the ameliorative effects of ethanol may lead to alcoholism especially in those symptomatic patients. As reported in most cases, ethanol of small doses could achieve the best therapeutic effects on patients of ethanol-responsive movement disorders. However, to maintain the same treatment effects, repeated doses are necessary and dose of ethanol increases over time (18). Therefore, alcoholism becomes a liability. Moreover, ethanol has various short-term and long-term adverse effects. With increasing frequency and rising doses due to tolerance, consumption of ethanol can cause irreversible damage to brains, livers, and other organs (93). Considering that some of ethanol-responsiveness diseases are characterized as early onset such as myoclonus dystonia, the damage once occurred may significantly affect patients' quality of life and life span.

Therefore, since ethanol is not a perfect treatment for patients of ethanol-responsive movement disorders, it is urgent to identify an alternative medication with fewer liabilities. Medicines that might meet the criteria are classified as follows (Table 2), and their potential therapeutic mechanisms are shown in the Figure 3. Treatment efficacy and deficiencies of these drugs are discussed.

**Table 2:** Potential medicines for ethanol-responsive movement disorders.

**Drug name**	**Mechanisms**	***t*_**max**_**	***t*_**1/2**_**	**Adverse effects**	**Effects on ERMDs**	**Clinical trials**
Sodium oxybate	GABA_B_R agonist	25–46 min	35–60 min	Feeling drunk, dizziness, headache, abuse, respiratory depression, seizures, coma	Improve myoclonic, dystonia and tremor symptoms, but with an 60% occurrence rate of adverse events	NCT03292458; NCT00598078; EUCTR2007-002222-30-FR
SAGE-217	Extra-synaptic GABAAR agonist	1 h	16–23 h	Sedation	Well-tolerated and achieve improvement of tremor symptoms	NCT02978781
Zonisamide	LVA Ca^2+^ channel blocker; GABA agonist	2–6 h	52–60 h	Headache, nausea, fatigue, sleepiness, and diarrhea	Well-tolerated and improvement in ET, MD and tardive dyskinesia	NCT00616343; NCT01806805
Perampanel	Highly-selective, non-competitive AMPAR antagonist	0.55–11 h	64.9–129 h	Dizziness, somnolence, fatigue and headache	Markedly exhibit anti-tremor effects on ET patients	NCT02668146
1-Octanol and octanoic acid	Unclear (could serve as a LVA Ca^2+^ channel blocker)	70 min*	83.5 min*	Headache, asthenia, lethargy, nausea, dry mouth, taste change, heartburn, bloating, and constipation	Well-tolerated and receive remarkable improvements on amplitude and frequency of tremor	NCT00001986; NCT00102596; NCT00848172; NCT01468948; NCT01864525

* *1-Octanol is transformed into octanoic acid soon after administration. Here lists pharmacokinetics of octanoic acid*.

*t_max_, The amount of time that a drug is present at the maximum concentration in serum; t_1/2_, The time required for one half of the total amount of a particular substance in a biological system to be degraded by biological processes when the rate of removal is nearly exponential*.

### GABAergic Drugs

There is a long history of the application of GABA modulators on ethanol-responsive movement disorders. Primidone, for instance, is the first-line therapy for ET which could reduce the amplitude of tremor by 70% (2). Patients with MD also received symptomatic improvements from benzodiazepine and primidone (94, 95). However, both drugs could bring up various irreversible adverse effects soon after regular intake, and longer-term survey reveals that approximately half of patients would discontinue consumption eventually due to tolerance or side effects. Such condition is possibly because of differences between the lesion locations and acting sites of these drugs. Recent clinical trials, as a result, focused more on relatively safe, efficient, concentric drugs. Here, newly experimental GABAergic drugs are exhibited.

#### Sodium Oxybate

Sodium oxybate is currently an approved medicine for narcolepsy in the United States and also used for intravenous anesthesia, ethanol withdrawal, and abstinence in Europe (96). It is the sodium salt form of γ-hydroxybutyric acid (GHB), a structural analog of GABA that interacts with GABA_B_ receptors. When ingested orally, it could be quickly absorbed and cross the blood–brain barrier, converted into GHB within the brain (97). It is proved to be an agonist of most GABA_B_ receptors as well (98), which also suggests that it might deliver a similar effect to ethanol.

Recent clinical trials showed that over half of patients with ethanol-responsive movement disorders achieved symptom improvement after administration of sodium oxybate, and the improvement of myoclonus at rest usually occurs earlier than that of myoclonus in action. In 2000, Priori et al. (99) reported a patient with ethanol-responsive MD whose myoclonus was improved with daytime dosing of sodium oxybate. Later on, patients with post-hypoxic myoclonus (96) and spasmodic dysphonia (100) (NCT03292458) were also reported to demonstrate dose-dependent improvements from sodium oxybate. In addition, Termsarasab and Frucht (20) reported that two patients of prostate cancer with ethanol-responsive action myoclonus of one leg went into remission after initiation of sodium oxybate.

However, a multiple-dose, double-blind, placebo-controlled study in ET patients (NCT00598078) did not receive an ideal result. Although the group using sodium oxybate 1.5 g at ~8 am, placebo at ~10 am, and sodium oxybate 1.5 g at ~12 pm appeared to get improvement in essential rating tremor scales, safety problems cannot be ignored that 60% of group members met with adverse events during the studies. Currently, a single- and multiple-dose study to compare the pharmacokinetics, pharmacodynamics, safety, and tolerability of sodium oxybate in subjects with moderate to severe ET (EUCTR2007-002222-30-FR) is under way, which might bring about a more convincing and comprehensive evaluation on this medicine.

Application of sodium oxybate is facing a number of problems. Firstly, the average time for sodium oxybate to peak plasma concentration ranges from 35 to 60 min, and it is rapidly eliminated through several steps into carbon dioxide and water with a terminal half-life of 36 ± 9 min for 25 mg/kg and 39 ± 7 min for 35 mg/kg (101). This means sodium oxybate could only serve for temporary improvement rather than long-term control. In addition, the best effect of the drug was observed in patients who reported improvement of symptoms with small doses of alcohol. For those patients less sensitive to ethanol, they might get benefits from sodium oxybate with increased doses (100), but adverse effects such as dizziness, headache, abuse, respiratory depression, seizures, and coma, especially when taken with other central nervous system depressants like ethanol, will overshadow the ethanol-mimetic effects (102). These side effects will not only limit the practical use of sodium oxybate but also bring up safety issues.

In short, sodium oxybate has potential in improving symptoms of ethanol-responsive movement disorders temporarily and rapidly, especially for patients with high sensitivity to ethanol, but its safety is a concern.

#### Neuroactive Steroids

Neuroactive steroids (NASs) are one of positive modulators for the δ subunit of extra-synaptic GABA_A_Rs as mentioned above (103). SAGE-547, the first-generation NAS, has completed an exploratory study in ET. Although well-tolerated, little evidence for increased efficacy was found during the open-label period, and increased sedation and sleepiness at the higher dose questioned its safety for long-term treatment (104). Subsequently, SAGE-217, another NAS, is under clinical trials for ET. A phase 1 single ascending dose (SAD) and multiple ascending dose (MAD) clinical trial has completed and a phase 2 clinical trial (NCT02978781) is currently underway. Results of the phase 1 study showed that SAGE-217 was orally bioavailable, with a terminal-phase half-life of 16–23 h and a tmax of ~1 h. No serious adverse events were reported during both SAD and MAD studies, and mild adverse events including sedation were dose-dependent and transient. The primary results for phase 2 research indicated that SAGE-217 30-mg capsules were generally well-tolerated and achieve improvement of tremor symptoms *via* Kinesia™ and TETRAS upper-limb combined kinetic and total scores (105). Detailed data is needed to fully assess the reliability and validity of this trials, and further randomized controlled trials (RCTs) should be carried out to fully explore the efficacy and safety of SAGE-217.

### LVA Ca^2+^ Channel Blockers

Little data are available on the effects of LVA Ca^2+^ channel blockers on ERMDs. To date, among all the definite LVA Ca^2+^ channel blockers, only ethosuximide and zonisamide were reported to have effects on animal models (74, 106, 107), but little clinical efficacy was found in ethosuximide (108). Here, progress on zonisamide is elaborated in clinical trials.

#### Zonisamide

Zonisamide is a medication mainly used to treat symptoms of epilepsy in the United States, United Kingdom, Japan, South Korea, and Australia with different application ranges and is also used in the treatment of motor symptoms of Parkinson's disease in some countries such as Japan (109). It has multiple mechanisms of action, including inhibition of sodium and T-type calcium channels, to suppress neuronal hypersynchronization (110) and modulation of GABAergic neurotransmission (111). Thus, zonisamide could mimic the influence of ethanol in different ways and is likely to replace ethanol for similar or even better results with fewer side effects.

Clinical evidence has demonstrated the potential of zonisamide for treatments of ethanol-responsive movement disorders. In 2008, Zesiewicz et al. (112) conducted a double-blind placebo-controlled trial to investigate the effects of zonisamide in patients of essential tremor (NCT00616343) and found that tremor amplitude was significantly improved in the group with zonisamide intake, and 60% of patients of this group demonstrated improvements while the others felt that their tremor was at least “minimally improved.” In 2012, Iwata et al. (113) discovered that zonisamide may be useful for the treatment of tardive dyskinesia, a disease reported to be responsive to ethanol. Patients of myoclonus dystonia also benefited from use of zonisamide (NCT01806805), with significant improvements in action myoclonus, myoclonus-related functional disability, and dystonia (114).

Zonisamide has a clear advantage over ethanol in that it has been proved relatively safe, effective, and well-tolerated in long-term treatments as monotherapy or adjunctive therapy (115, 116). Additionally, zonisamide has a pharmacokinetic characteristic favorable for clinical use. It is rapidly absorbed orally, with a bioavailability close to 100%. The time to peak blood levels is achieved in about 2–6 h (117), with the half-life of 52–60 h found in single-dose studies (118, 119). Therefore, zonisamide can be used in conjunction with ethanol or sodium oxybate that the former one serves as a long-term control; the latter two can work for temporary relief. Nevertheless, zonisamide is associated with some common adverse effects of this medicine, such as headache, nausea, fatigue, sleepiness, and diarrhea (117). These side effects will definitely impact the quality of life in patients, reduce compliance, and even cause irreversible damages. In addition, all the clinical trials we found were small in sample size and poor at blinding, which is insufficient for clinical applications. RCTs with adequate methodology and large samples should be performed to assess long-term efficacy and safety.

### Glutamate Receptor Blockers

Although glutamatergic pathways are considered tightly associated with ERMDs, not much clinical trial has been done on corresponding drugs. Here we introduce one potent blocker for glutamate receptors.

#### Perampanel

Perampanel, approved as an antiepileptic drug currently, is a highly selective, non-competitive AMPAR antagonist that could regulate glutamate neurotransmission. Perampanel was rapidly absorbed when taken orally, reaching its maximum concentration after 0.55–11 h, and its terminal-phase half-life was 64.9–129 h, indicating its rapid onset and long maintenance (120). The most common adverse effects for perampanel are dizziness, somnolence, fatigue, and headache (121). An open-label trial of perampanel has completed for ET treatment (NCT02668146). In a small sample size of 12, perampanel exhibited marked anti-tremor effect (122). RCTs with large samples and long-term follow-up are still needed to confirm its effectiveness and safety.

### Long-Chain Alcohols and Their Ramifications

Till now, it remains uncertain whether or not using one or some of the drugs mentioned above could completely mimic the effects of ethanol in all aspects, especially considering the uncertain involvement of those unknown mechanisms. Thus, other alcohols were expected to substitute ethanol in a better way. Alcohols ranging from methanol to decanol were all studied *in vitro*, in which octanol, unlike others, showed its ability to block LVA Ca^2+^ channels, and 1-octanol exhibited the best efficacy mainly due to its longer duration among all isoforms in models of essential tremor (123). Still, 1-octanol might act through other mechanisms like GABA-receptor interaction.

#### 1-Octanol and Octanoic Acid

1-Octanol is approved as a food flavoring substance and a precursor to perfumes. It is rapidly converted to octanoic acid after administration. Octanoic acid is then likely to work as active metabolite in the body, while the concentration of 1-octanol is maintained at a relevantly low level (124). In 1989, Sinton et al. (125) revealed that 1-octanol was an effective blocker of harmaline-induced tremor. Bushara et al. (126) then conducted a pilot trial of 1-octanol in ET patients (NCT00001986) and found that 1-octanol significantly decreased tremor amplitude for up to 90 min and no severe side effects or signs of intoxication were observed at a single oral dose of 1 mg/kg. A dose-escalation study (NCT00102596) of oral 1-octanol in patients with essential tremor then demonstrated that 1-octanol was well-tolerated up to 64 mg/kg without overt intoxication, and higher doses may produce more sustained benefit (127).

Octanoic acid is also used as food and cosmetic additive and sometimes used in assessment of gastric emptying. It could permeate the blood–brain barrier in the rat by a rate of 94% (128). In 2012, a study in a harmaline-induced mouse model of ET showed that octanoic acid could suppress tremor following dose-dependent efficacy (129). To assess an oral, single, low dose of octanoic acid in patients with ethanol-responsive ET, Haubenberger et al. (130) conducted a randomized controlled study (NCT00848172) and reached a conclusion that octanoic acid was effective after 180 min of intake. Furthermore, Voller et al. (131) described the dose-dependent effect of octanoic acid in patients with essential tremor (NCT01468948); a single dose of 128 mg/kg was not associated with serious adverse events. Patients with essential voice tremor also received considerable improvement on magnitude of amplitude and frequency tremor (NCT01864525), further supporting the potential utility of octanoic acid for ERMDs (132).

One overt advantage of 1-octanol and octanoic acid is its safety. Based on the results of all the clinical trials so far, mild adverse effects include headache, asthenia, lethargy, nausea, dry mouth, taste change, heartburn, bloating, and constipation (130). Moreover, among all the research, the maximum tolerated doses of two drugs are still unknown, further highlighting their safety. In addition, 1-octanol and octanoic acid show a longer duration than ethanol. Octanoic acid, serving as one possible drug as well as being the primary metabolite of 1-octanol, has a half-life time of 83.5 min (130). Nevertheless, the effects of long-chain alcohol and their ramifications on other ethanol-responsive movement disorders require additional experimental and clinical investigations. In other movement disorders, 1-octanol and octanoic acid may not show the best efficacy.

## Limitations

The present understanding on the mechanisms and pharmacotherapy of ERMDs should be considered in the context of the following limitations.

In the aspects of neural networks involved in these diseases, though compelling evidence has supported the essential role of the cerebellum and circuits linked to the cerebellum, the unsolved question is whether these movement disorders are originated from the cerebellum, or are subsequently mediated by other structures within the shared anatomical networks, or are the result of disruption in connectivity between several brain structures. Future investigations on this question will be helpful for a better understanding of these diseases and development of optimal pharmacotherapy and neurosurgical intervention for ERMDs.

As for pathophysiologic mechanisms, the current hypothesis can only partly elucidate the effects of ethanol on ERMDs. It has been proved that genetic etiology is probably an indicator of ethanol responsiveness (21, 133). Early-onset ET has a higher possibility of a positive family history, and Hopfner et al. (133) compared patients under 24 years of age to those over 46 of ET and found that ethanol responsiveness was much more common among young patients. Junker et al. (21) recently concluded in a recent large sample research that ethanol responsiveness of dystonia is associated with a positive family history for movement disorders, generalized dystonia, and an earlier age at onset. These two studies suggest that patients who have an underlying genetic contribution are more likely to respond beneficially to ethanol. However, the genetic connection among GABA systems and ERMDs, though important, remains unclear. On the one hand, it suggests that during genetic analysis of ERMDs, genes related to GABA pathways need to be paid more attention to. On the other hand, based on discovered genetic risk factors, some new mechanisms might be uncovered for a better understanding and more effective therapy for ERMDs. In addition, some deficiencies, especially safety concerns, exist among alternative medicines mentioned above. Lack of large-sample, long-term clinical trials also make it insufficient for clinical application of these medicines. Therefore, developing a more effective and safer drug is key to further development of treatment for diseases responsive to ethanol, which is possibly inspired by deeper understanding of ethanol responsiveness.

## Conclusion

Ethanol-responsive movement disorders are a group of dyskinesia, of which clinical manifestation could receive significant improvement after consumption of ethanol. Despite their various clinical features, these diseases share similar anatomical targets and common physiopathological sites for ethanol. Cerebellum and cerebellum-related neural circuits are the most potent common anatomical regions involved in ERMDs, in which GABA pathways, LVA Ca^2+^ channels, and glutamatergic system play key roles. Corresponding drugs have received clinical results with fewer liabilities compared to ethanol, including GABAergic drugs like sodium oxybate and NASs, LVA Ca^2+^ channel blockers like zonisamide, glutamate receptor antagonists like perampanel, and long-chain alcohols like 1-octanol and its corresponding acid. Promoting the use of these drugs may be a boon to patients, improving their quality of life and extending their lives. However, there is still a long way for clinical application of these drugs due to lack of large-sample, long-term follow-up data. Further exploration on neuro-circuits and mechanisms underlying ethanol responsiveness will also deepen the understanding of these diseases and accelerate the discovery of ideal treatment.

## Author Contributions

JW was the major contributor for manuscript writing. HT and SC contributed to manuscript critique and revision. LC was a contributor in funding, manuscript critique, and revision. All authors read and approved the final manuscript.

## Conflict of Interest

The authors declare that the research was conducted in the absence of any commercial or financial relationships that could be construed as a potential conflict of interest.
